# Spectroscopic Studies on the Molecular Ageing of Serum Albumin

**DOI:** 10.3390/molecules22010034

**Published:** 2016-12-27

**Authors:** Mariola Chudzik, Małgorzata Maciążek-Jurczyk, Bartosz Pawełczak, Anna Sułkowska

**Affiliations:** School of Pharmacy with the Division of Laboratory Medicine in Sosnowiec, Medical University of Silesia, Chair and Department of Physical Pharmacy, Jagiellońska 4, 41-200 Sosnowiec, Poland; mmaciazek@sum.edu.pl (M.M.-J.); bpawelczak@sum.edu.pl (B.P.); asulkowska@sum.edu.pl (A.S.)

**Keywords:** molecular ageing, serum albumin, metoprolol, second derivative, circular dichroism

## Abstract

Pathological states in the organism, e.g., renal or hepatic diseases, cataract, dysfunction of coronary artery, diabetes mellitus, and also intensive workout, induce the structural modification of proteins called molecular ageing or N-A isomerization. The aim of this study was to analyze the structural changes of serum albumin caused by alkaline ageing using absorption, spectrofluorescence, and circular dichroism spectroscopy. The N-A isomerization generates significant changes in bovine (BSA) and human (HSA) serum albumin subdomains—the greatest changes were observed close to the tryptophanyl (Trp) and tyrosyl (Tyr) residue regions while a smaller change was observed in phenyloalanine (Phe) environment. Moreover, the changes in the polarity of the Trp neighborhood as well as the impact of the ageing process on α-helix, β-sheet content, and albumin molecule rotation degree have been analyzed. Based on the spectrofluorescence study, the alterations in metoprolol binding affinity to the specific sites that increase the toxicity of the drug were investigated.

## 1. Introduction

Serum albumin is the most important carrier protein for numerous endogenous and exogenous ligands [1]. Human serum albumin (HSA), which contains 585 amino acids, has a single tryptophanyl residue (Trp214 located in subdomain IIA) while bovine serum albumin (BSA), which contains 582 amino acids, has two tryptophanyl residues (Trp214 and Trp135 located in subdomains IIA and IB, respectively) [1,2]. Due to the changes in pH, the following serum albumin isomerization are formed: N(normal)-F(fast) isomerization at pH between 5.0 and 3.0, F(fast)-E(expanded) isomerization below pH 3.5, N(normal)-B(basic) isomerization at pH between 7.0 and 9.0 [3], and N(normal)-A(aged) isomerization and A(aged) form of albumin at pH 9.0. The N(normal)-A(aged) isomerization leads to the structural relaxation of the protein structure. It mainly affects the N-terminal region and thereby may influence the ligand binding affinity [4,5]. The A(aged) form of bovine serum albumin (ABSA) was first described by Nikkel and Foster [6]. They observed that ABSA demonstrates a different charge, solubility and optical properties in comparison to that of non-modified BSA. They also noticed that the A isoform has a permutation of disulfide bonds which does not allow for the fold into the non-modified conformation at lower pH.

In this paper, the conformational changes of HSA and BSA have been investigated using absorption (ultraviolet–visible UV-Vis), spectrofluorescence (SFM), and circular dichroism (CD) spectroscopy. Due to the presence of two tryptophanyl residues, the study of BSA enabled the observation of the changes not only in the subdomain IIA (Trp214) but also in the subdomain IB (Trp135). The use of a second derivative of absorption and fluorescence spectroscopy allowed us to demonstrate that these methods are appropriate for describing and monitoring the changes that occur in the environment of the protein aromatic residues (phenyloalanine (Phe), tyrosine (Tyr), and tryptophan (Trp). Since the value of the derivative is not only dependent on the absorbance but also on the shape of the primary spectrum, derivative spectroscopy is more sensitive and selective than classic spectroscopy. The overlapping bands in the primary spectrum are separated into the derivative spectrum during the differentiation of the zero-order spectra [7,8]. The absorption spectrum of the protein is a sum of physical absorption and light scattering while the second derivative effectively eliminates the effect derived from the light scattering and the spectral contribution of the disulphide bonds [7]. Secondary structure of the aged human and bovine serum albumin was characterized by CD spectroscopy in the far-UV spectral region (from 200 nm to 250 nm).

**Scheme 1:**
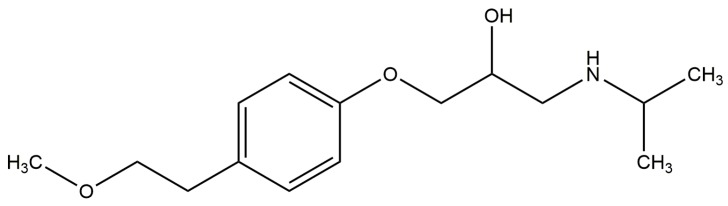
Chemical structure of metoprolol (MET).

We also performed detailed analysis of metoprolol (MET) interaction with N and A forms of human and bovine serum albumin using fluorescence spectroscopy. Computational simulation has been used in order to identify MET binding sites on the protein. Metoprolol (MET, 1-Isopropylamino)-3-[4-(2-methoxyethyl)phenoxy]propan-2-ol, Scheme 1) is a cardio selective β-blocker, used in the treatment of hypertension, angina pectoris, arrhythmia, myocardial infarction, and heart failure [9]. 

The binding of MET to HSA and BSA isomers (N and A) provides useful information about its pharmacological effect because the content of the MET fraction can be modified as a result of the interaction with serum albumin. 

## 2. Result and Discussion

### 2.1. Absorption and Fluorescence Characteristic of Aged Serum Albumin—Second Derivative

**Figure 1:**
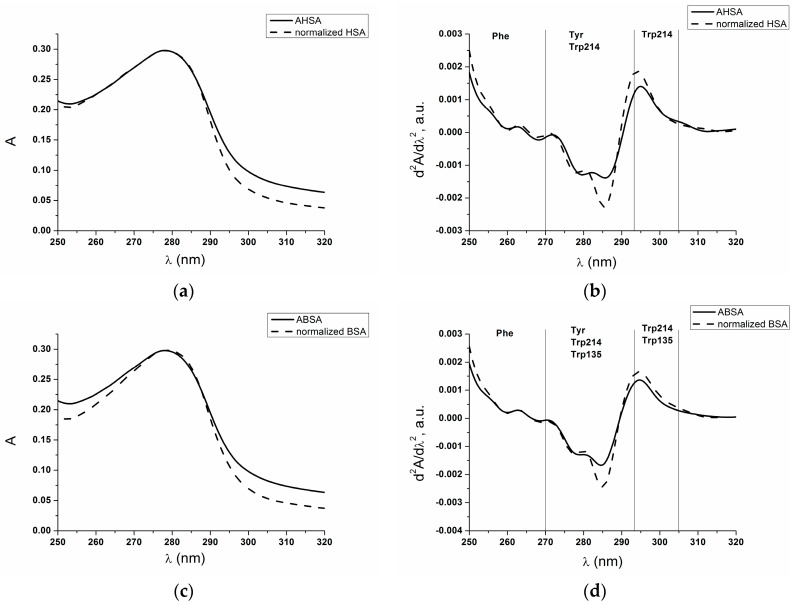
Normalized zero-order (**a**,**c**) and second derivative absorption spectra (**b**,**d**) of non-modified and aged human serum albumin (HSA and AHSA, respectively) and bovine serum albumin (BSA, and ABSA, respectively) ; protein concentration was 5 × 10^−6^ mol/dm^3^.

The normalized absorbance spectra of aged human (HSA) and bovine (BSA) serum albumin in phenylalanine (Phe), tyrosine (Tyr), and tryptophans (Trp) regions are shown in Figure 1a,c. Figure 1b,d presents their second derivatives.

A contribution of the protein aromatic residues, phenylalanine (Phe), tyrosyl (Tyr), and tryptophanyl (Trp), has been determined using derivative spectroscopy. It is noteworthy that the contribution of (Phe), (Tyr), and (Trp) residues in the total protein absorbance cannot be determine using the method of zero-order spectrum because molar absorption coefficients of Phe, Tyr, and Trp differ significantly. These values were obtained by Mihályi and are equal to: $\varepsilon_{Phe}^{248}$ = 110.6 dm^3^·mol^−1^·cm^−1^, $\varepsilon_{Tyr}^{276}$ = 1330 dm^3^·mol^−1^·cm^−1^, and $\varepsilon_{Trp}^{281}$ = 5620 dm^3^·mol^−1^·cm^−1^ [10]. The first derivative spectra dA/dλ is a rate of the absorbance (A) which changes with the change of the wavelength (λ), while the second derivative dA^2^/dλ^2^ is the rate at which this change occurs. As it was reported by Kuś [8] and Karpińska [11], subtle structures (arms, inflection, size of descent angle of spectrum), faintly visible in the zero-order absorption spectrum become more pronounced in the second derivative spectrum. Figure 1a,c present the zero-order spectra of HSA and BSA, arithmetically normalized to the maximum absorbance of the aged HSA (AHSA) and ABSA. In order to show subtle changes of albumin’s tertiary structure due to the ageing process, the second derivative spectra have been shown in Figure 1b,d. The second derivative absorption spectra of HSA, AHSA, BSA, and ABSA allow us to distinguish albumin chromophores. The spectral wavelength range from 250 nm to 270 nm relates to phenylalanine (Phe) residues and the participation of other aromatic chromophores in this wavelength range is negligible. A wavelength range from 293 nm to 305 nm relates only to the tryptophanyl (Trp) residues and the wavelength range between 270 nm and 293 nm relates to tyrosyl (Tyr) and tryptophanyl residues (Trp214 in HSA and BSA and Trp135 in BSA) [12,13].

From the second derivative absorption spectra, the d^2^A/dλ^2^ values of AHSA, normalized HSA, BSA, and normalized BSA were determined and collected in Table 1.

Qualitative analysis (Figure 1) and the second derivative values of the analyzed spectra (Table 1) indicate that the ageing of human and bovine serum albumin leads to the alteration of the tertiary structure around Phe, Tyr, Trp214 (HSA), and Trp135 (BSA) residues. Human serum albumin (HSA) contains 31 phenyloalanine (Phe) residues located in each of six albumin subdomains and bovine serum albumin (BSA) contains 27. Eighteen Tyr residues of HSA and 19 of BSA are deployed in five subdomains of albumin (except subdomain IIIB) [1]. Therefore, it can be supposed that the structural changes caused by the ageing process refer not only to molecule fragments but also to whole albumin. The second derivative values obtained for aromatic amino acids residues (Trp, Tyr, Phe) are lower for both AHSA and ABSA structures and have been collected in Table 1. 

**Figure 2:**
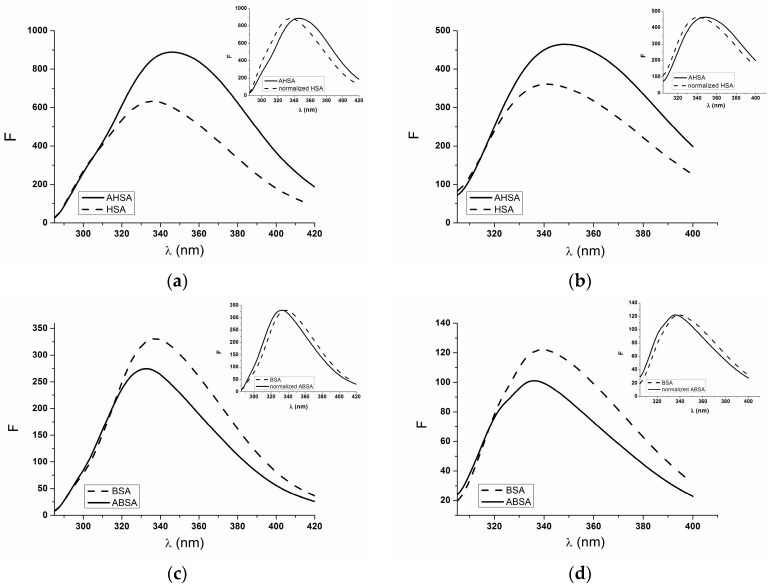
Fluorescence emission spectra of non-modified (HSA, BSA) and aged (AHSA, ABSA) human and bovine serum albumin excited at 275 nm (**a**,**c**) and 295 nm (**b**,**d**); in the insert: the spectra normalized to their respective maxima around 340 nm.

As it was described by Kagan et al. [14] and Ankaru et al. [15], during the ageing process, the aromatic (tryptophan, tyrosine, phenylalanine) and basic (cysteine, methionine, lysine, arginine, histidine, proline) amino acids are oxidized since they are more sensitive to proteolysis than other amino acids. The formation of oxidized products as a result of ageing causes the changes in the function of albumin, for example, the changes of metal ions binding (Cu^2+^, Co^2+^, Ni^2+^), the reduction of antioxidant properties, and the reduction of thioesterase activity (Trp residue is oxidized). Furthermore, the ageing process causes the tyrosine oxidation and the generation of tyrosine radicals, which form the crosslinks and aggregates with the same or different molecules. Varshavsky [16], in his paper, described the loss of serum albumin chaperone-like activity that cause the accumulation of modified protein leading to apoptosis or necrosis. Eldeeb and Fahlman [17,18,19], in their scientific works, demonstrated anti-apoptopic roles for the N-end rule pathway. They also presented that degradation can be modified by changing the identity of the N-terminal amino acids. They noticed this phenomenon during the investigation the proteolytical degradation of Lyn tyrosine and bone marrow kinases by the N-rule machinery [17,19]. Emission fluorescence spectra were obtained by the use of two excitation-wavelengths: λ_ex_ 275 nm and 295 nm. When serum albumin is excited at λ_ex_ 295 nm, tryptophanyl residues are responsible for all albumin fluorescence, and tyrosyl residue fluorescence is insignificant. When the protein is excited at λ_ex_ 275 nm, the fluorescence from both tryptophanyl and tyrosyl residues of albumin is observed [20]. Figure 2 illustrates emission fluorescence spectra of non-modified (HSA, BSA) and aged (AHSA, ABSA) human and bovine serum albumin at excitation wavelengths of 275 nm (a), (c) and 295 nm (b), (d). In the inserts of Figure 2, the spectra normalized to their respective maxima around 340 nm are shown.

**Figure 3:**
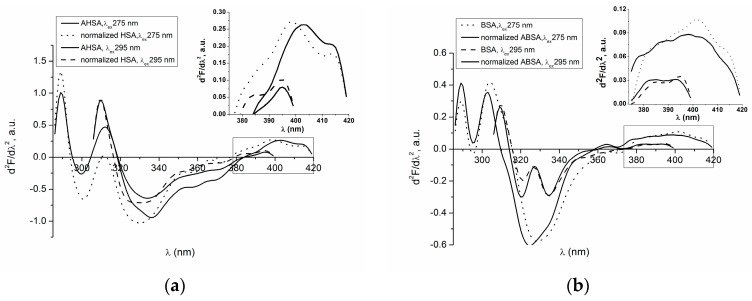
Second derivative fluorescence spectra of normalized and aged isomers of (**a**) human (HSA) and (**b**) bovine (BSA) serum albumin excited at 275 nm and 295 nm, in the insert: second derivative fluorescence spectra of albumin (λ > 370 nm) derived from Trp residue(s).

For aged human serum albumin (AHSA) the increase in fluorescence intensity is observed in comparison to non-modified HSA, while the fluorescence intensity obtained for non-modified bovine serum albumin (BSA) is higher in comparison to aged BSA. Before the determination of the second derivative spectra, the fluorescence spectra of non-modified HSA were normalized to AHSA fluorescence spectra and ABSA fluorescence spectra were normalized to non-modified BSA fluorescence spectra (Figure 2, in the insert). Second derivative spectra of non-modified (HSA, BSA) and aged (AHSA, ABSA) human and bovine serum albumin normalized to their respective maxima are presented in Figure 3. 

Batochromic shift of second derivative fluorescence spectra of tryptophanyl residue (Trp214) of non-modified (HSA) and aged (AHSA) human serum albumin is observed at λ_ex_ 275 nm (Figure 3a, in the insert). The peaks maxima of the second derivative fluorescence spectra of HSA tryptophanyl residue (Trp214) appear at 398 nm and 415 nm and for AHSA at 403 nm and 414 nm. The valley in HSA and AHSA second derivative fluorescence spectra occurs at 408 nm and 411 nm, respectively (Figure 3a, in the insert). Because bathochromic shift was observed in both maxima, valley, and shoulders it, means that the aged human albumin environment around Trp214 becomes more polar. It confirmed our previous conclusions obtained from the analysis of the fluorescence emission spectra that the ageing process of HSA causes an increase in polarity in subdomain IIA (Figure 2a, in the insert). 

Second derivative fluorescence spectra of HSA tryptophanyl residue (Trp214) excited at λ_ex_ 295 nm exhibit two peaks maxima (383 nm and 395 nm) and mark valley at 389 nm. While second derivative spectra of AHSA tryptophanyl residue (Trp214) have only one peak maximum (395 nm) and small shoulder from the blue side of the peak. The ageing of HSA does not cause the shift of the second derivative fluorescence spectrum (Figure 3a, in the insert).

In comparison to non-modified BSA (Figure 3b, in the insert) and in opposition to HSA (Figure 3a, in the insert), the second derivative fluorescence spectra of aged BSA (λ_ex_ 275 nm) is blue shifted. This phenomenon is caused by the Trp135 located not only in the BSA subdomain IB but also in the environment containing charged residues. At the excitation wavelength λ_ex_ 275 nm, second derivative fluorescence spectra of non-modified bovine serum albumin (BSA) tryptophanyl residues (Trp214, Trp135) exhibit two maxima (at wavelengths 402 nm and 413 nm) and three shoulders from the blue side of the peak. Aged BSA (ABSA) has one maximum at 398 nm and a shoulder from the red side of the peak instead of the second peak observed for non-modified BSA. In the ABSA second derivative fluorescence spectra, two shoulders from the blue side are observed (Figure 3b, in the insert). 

Two maxima of both second derivative fluorescence spectra, BSA and ABSA, are observed at excitation wavelength λ_ex_ 295 nm: for BSA at wavelengths 384 nm and 395 nm and for ABSA at wavelengths 383 nm and 393 nm. It means that the second derivative fluorescence spectra of aged bovine serum albumin (ABSA) is blue-shifted. It is noteworthy that the second derivative fluorescence spectra values of HSA and BSA tryptophanyl residues are higher in comparison to that of AHSA and ABSA (Figure 3, in the inserts). The same effect was obtained using UV technique (Table 1).

The values of the second derivative fluorescence spectra were determined by *peak to peak* method, where the distance from the positive peak maximum to the negative peak minimum is measured [7,21]. Mozo-Villarías [22] defines the *peak to peak* method of the derivative value as an empirical parameter *H* (*relative peak* *composition*). For the excitation λ_ex_ 295 nm, parameter *H* is the difference between minimum and maximum in the region between 370 nm and 400 nm of the second derivative spectra. Whereas for the excitation λ_ex_ 275 nm, parameter *H* is the difference between the minimum and maximum below the wavelength 320 nm of the second derivative spectra. The empirical parameter *H* is an indicator of the polarity in the tryptophanyl (Trp214 in HSA, Trp214, and Trp135 in BSA) and tyrosyl (Tyr) residues environment [21,22]. The changes in the second derivative value in the wavelength range between 370 nm and 400 nm (the red-side of the absorption spectrum) point to the structure reorganization around tryptophanyl residues in HSA (Trp214) and BSA (Trp214, Trp135). The changes of the second derivative value in the wavelength range below 320 nm indicate the rearrangement of the HSA and BSA structures mainly around tyrosyl residues (Tyr). The values of parameter *H* are collected in Table 2.

The decrease in polarity around tyrosyl residues of aged human (AHSA) and bovine (ABSA) serum albumin results in the blue-shift of second derivative fluorescence spectra at excitation λ_ex_ 275 nm (the value of parameter *H* increases) (Table 2, Figure 3). The differences in polarity occurring at the ageing process are greater for HSA than BSA. 

At λ_ex_ 295 nm, the increase in polarity around aged human serum albumin tryptophanyl residue (Trp-214) was observed (the value of parameter *H* decreases). Mozo-Vilarias [22] determined the value of the HSA relative peak composition value (λ_ex_ 295 nm) and demonstrated that the environment of tryptophanyl residue (Trp214) is rather hydrophobic. It was confirmed by Kosa et al. [23]. The structural changes of human albumin N-B isomerization observed by the spectroscopic techniques (circular dichroism, fluorescence, ^1^H-NMR) and differential scanning calorimetry (DSC) indicated that Trp residue is buried in a hydrophobic environment and fluctuations of the residue increased with the increase of pH (Trp residue is exposed to polar environment). The increase in polarity around the Trp214 of aged HSA can reduce the effective solubility of those hydrophobic drugs bound in the subdomain IIA of albumin and influence its physiological activity. Oettl and Stauber [24] demonstrated that both ageing and in the presence of diseases influence the redox state of albumin which results in changes of ligand binding (i.e., some non-steroidal anti-inflammatory drugs, calcium channel blocker, benzodiazepine group) to serum albumin. 

The ageing of BSA causes the decrease in polarity around the tryptophanyl residues (Trp-214 and Trp-135) (λ_ex_ 295 nm), but the differences are negligible in comparison to that of BSA.

The modification of basic amino acids such as lysine and arginine influenced significantly on the hydrophobicity and net charge of albumin [25]. Moreover, Varshavsky [16] observed shortened HSA and BSA half-life due to the alterations of the N-terminal lysine and histidine, which are modified during ageing. It is possible that HSA and BSA are degraded by the N-end rule pathway [26]. 

### 2.2. Circular Dichroism (CD) Characteristic of Aged Serum Albumin

**Figure 4:**
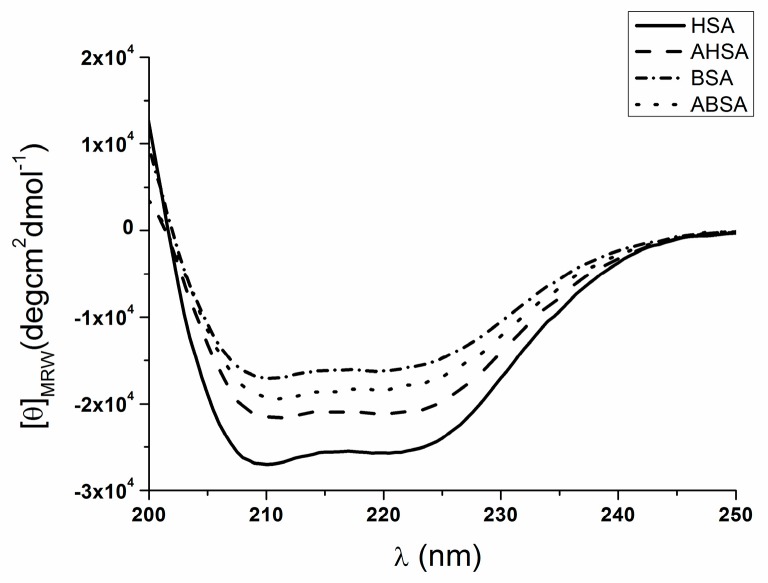
Far—ultra-violet circular dichroism (UV CD) spectra of native and aged human (HSA, AHSA) and bovine (BSA, ABSA) serum albumin; protein concentration was 1 × 10^−6^ mol/dm^3^.

In order to observe how the protein secondary structure changes during ageing, far-UV CD spectroscopy was used. The solutions of A and N isoform of HSA and BSA were scanned within the wavelength range between 200 nm and 250 nm. A spectrum for each of the analyzed proteins in the non-modified and aged form is shown in Figure 4. The percentage content of the secondary structure elements of HSA, AHSA, BSA, and ABSA are collected in Table 3.

The CD spectra of HSA, AHSA, BSA, and ABSA have two negative bands in the UV region at 209 nm and 222 nm, which are characteristic of an α-helical structure of protein (Figure 4). The ageing process changes the percentage (%) content of the albumins secondary structure elements. Hazra and Kumar [27] claimed that both bands are assigned to the transition of the peptide bond of α-helical structure. AHSA CD spectrum shows a decrease in the ellipticity at 222 nm, whereas the ABSA ellipticity increases in comparison to that of the non-modified HSA and BSA (Figure 4, Table 3). Dockal et al. [28] demonstrated that the alteration in ellipticity is accompanied by the destabilization of secondary structure, and it also indicated a reduction of α-helical content. In our study, the N-A isomerization of bovine and human serum albumin caused the reduction of α-helical content. For aged HSA (AHSA), ~16.5% of α-helical content reduction was observed, while for aged BSA (ABSA), only ~4.5% was observed. The remaining secondary structure elements caused stronger alterations of HSA than BSA (Table 3). It indicates that the perturbation of secondary structures are more significant in human than bovine serum albumin (Table 3). Era et al. [29] received the A-isoform of BSA by S-S (disulfide bridges) exchange reaction and concluded that the aged structure of albumin is stabilized covalently by disulfide bonds. This stabilization causes an alteration of the A form, resulting in the decrease in the helical content and the increase in hydrodynamic volume. The thiol/disulfide exchange reaction at alkaline pH values causes the production of aggregates of small dimension. When the pH of the solution is far away from the protein pI, the net charge increases and the formation of aggregate structures is observed. Based on the reduction of *α*-helical content and the increase of β structural elements of both AHSA and ABSA (Table 3), the formation of aggregates has been identified. In elderly people and during the diseases associated with age, the accumulation of modified proteins due to the formation of crosslinks and aggregates occurs [30,31], and protein becomes more resistant to intracellular proteolytic systems [32].

### 2.3. Effect of N-A Isomerization on Metoprolol (MET) Binding to Serum Albumin

**Figure 5:**
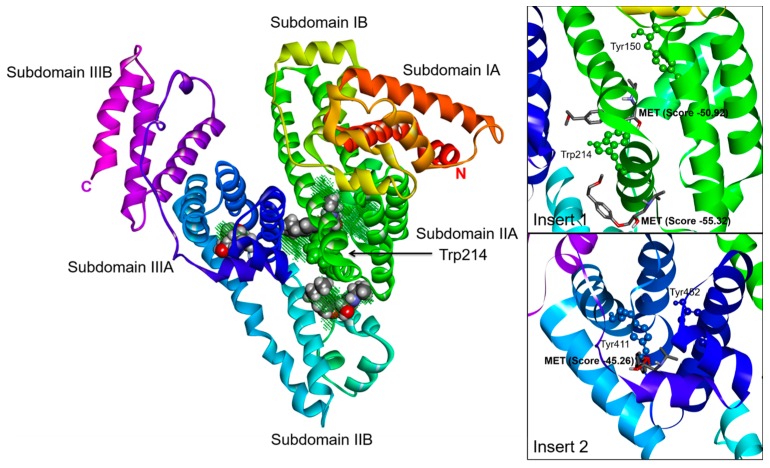
Computational structure of HSA (PDB 1AO6) complexed with metoprolol (METs). In the inserts: molecules of MET in subdomains IIA (insert 1) and IIIA (insert 2).

**Figure 6:**
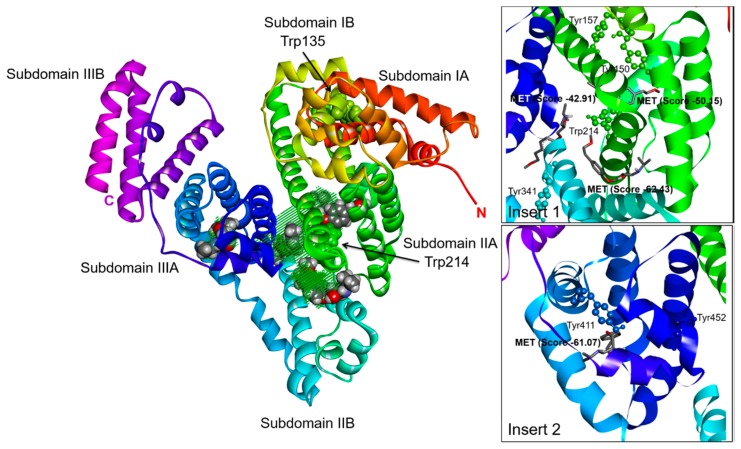
Computational structure of BSA (PDB 4F5S) complexed with METs. In the inserts: molecules of MET in subdomains IIA (insert 1) and IIIA (insert 2).

There are two high affinity binding sites in the HSA and BSA tertiary structure for exogenous and endogenous ligands. According to Sudlow’s nomenclature, site I includes six helices of subdomains IIA and the loop associated with subdomain IB. Site II is much smaller than site I and includes six helices of subdomain IIIA [33,34]. As a confirmation of MET binding to I and II, the binding sites of HSA and BSA crystal structures have been created using computational simulation. Figure 5 and Figure 6 presents the mechanism of MET binding to the fluorophores of the analyzed proteins.

**Figure 7:**
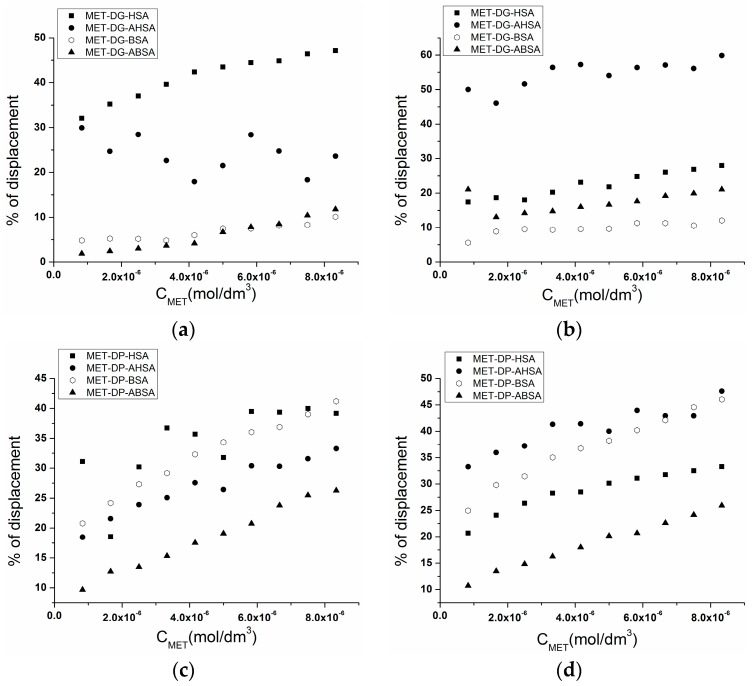
Displacement percentage of dansylglicyne (DG) and dansyl-l-proline (DP) from serum albumin (SA) by metoprolol (MET). [SA]:[DG] molar ratio 1:0.5 (**a**); 1:1 (**b**); [SA]:[DP] molar ratio 1:0.5 (**c**,**d**). DG and DP stock concentrations were 5 × 10^−4^ mol/dm^3^, [SA] concentration was 1 × 10^−6^ mol/dm^3^.

In order to determine the binding affinity of metoprolol (MET) to specific binding sites located in subdomains IIA and IIIA of the HSA and BSA tertiary structure, the method of fluorescent probes (dansyl glicyne (DG) and dansyl-l-proline (DP)) was used. Dansyl glicyne (DG) was used as a marker for Sudlow’s binding site I (subdomain IIA), while dansyl-l-proline (DP) was used for Sudlow’s binding site II (subdomain IIIA). Fluorescence of DG and DP in aqueous solutions is only slight, while in the complex with albumin increases [35]. A displacement of DG and DP from their binding site in non-modified human and bovine serum albumin by MET was studied by the determination of displacement percentage of bound DG and DP. Moreover, the influence of the ageing process on displacement percentage of bound DG and DP from Sudlow’s site I and II by MET was conducted. In this way, it was possible to determine the specificity and differences in MET binding affinity to the specific sites of non-modified HSA and BSA and aged AHSA and ABSA serum albumins. Figure 7 presents the percentage of DG and DP displacement from their binding site in HSA, AHSA, BSA, and ABSA by MET at the albumin:MET molar ratio of 1:10, and the values of the percentage of displacement are collected in Table 4. 

In the presence of metoprolol (MET), the fluorescence intensity of all DG-albumin (DG-HSA, DG-AHSA, DG-BSA, DG-ABSA) and DP-albumin complexes (DP-HSA, DP-AHSA, DP-BSA, DP-ABSA) was decreased and a displacement of bound to albumin dansyl glicyne (DG) and dansyl-l-proline (DP) was registered. The displacement percentage of probes (DG, DP) from non-modified HSA binding sites (DG from site I and DP from site II) by metoprolol (MET) was similar and equals 47.12% and 39.16% at HSA:DG and HSA:DP, respectively, with a molar ratio of 1:0.5. At the molar ratio of HSA to both DG and DP at 1:1, the displacement percentage of DG and DP was reduced to 27.9% and 33.29%, respectively (Table 4). It can be concluded that the binding site of MET is located in the subdomains IIA and IIIA of the non-modified HSA structure and these results from in vitro experiments are consistent with the data obtained based on the computational simulations with the use of the Piecewise Linear Potential Protein-Ligand ANT System (PLANTS_PLP_) algorithm. 

The percentage of DG displacement from non-modified BSA by MET is low and did not exceed 13.0% at both molar ratios of BSA:DG at 1:0.5 and 1:1, while the calculated percentage of DP displacement from non-modified BSA equals 41.20% and 46.05% at BSA:DP molar ratios 1:0.5 and 1:1, respectively (Table 4). It indicates that MET also binds to subdomain IIIA of non-modified BSA.

The binding affinity of HSA site I has been changed by the alkaline ageing. At the molar ratio AHSA:DG of 1:0.5, the percentage of DG displacement decreased two times (23.60%), while at the molar ratio AHSA:DG of 1:1, it increased two times (Table 4). 

The serum albumin structure binding site I is large enough to be divided into three regions: region Ia, Ib, and Ic corresponding to warfarin, azapropazone, and n-butyl p-ABE, respectively [36]. Moreover, site I has been described as an “elongated sock shaped pocket” with hydrophobic foot and hydrophilic leg regions [36]. In our manuscript, we demonstrated that the alkaline ageing causes the increase in a polarity in subdomain II that results in DG binding to another region (Ia, Ib, Ic) of aged albumin in comparison to non-modified HSA.

At the molar ratio albumin:DP of 1:0.5, MET displaces DP from site II of aged HSA (33.28%) and non-modified HSA (39.16%). At the molar ratio albumin:DP of 1:1, the displacement of DP by MET from site II of AHSA is more effective (47.59%) in comparison to that of HSA (33.29%). This phenomenon indicates that the binding affinity of subdomain IIIA increases during HSA alkaline ageing. Moreover, the molar ratio AHSA:DG and DP of 1:1 should be used because site I involves three regions: Ia, Ib, Ic. Concluding, MET has greater binding affinity to aged than to non-modified HSA. The similar phenomenon was described by Chudzik et al. [37] for doxazosin binding affinity. 

On the contrary to AHSA, at both albumin:probe molar ratios of 1:0.5 and 1:1, MET displaces DP from site II of aged BSA (ABSA) less effectively than from non-modified BSA (Table 4). The binding of MET to site II involves helices: h1, h2, h3, h4 and h5. Because the amino acid sequence of these HSA and BSA helices are different in at least 18 positions, therefore, the alkaline ageing of HSA and BSA influences the metoprolol binding affinity in site II differently. 

Based on the conducted experiment, we observed that the increase of HSA elimination is caused by not only the basic amino acid modification but also the ageing influence on the specific albumin binding sites.

## 3. Materials and Methods

### 3.1. Chemicals

Human serum albumin (HSA, lot no. R27783) and bovine serum albumin (BSA, lot no. 056K0724) were obtained from MP Biomedicals and Sigma-Aldrich, respectively. Metoprolol (1-Isopropyloamino-3-[4-(2-methoxyethyl)phenoxy]propan-2-ol, MET), dansylglycine (DG) and dansyl-l-proline (dDP) were purchased from Sigma-Aldrich (Darmstadt, Germany). TRIS, HCl, glycine (Gly) and NaOH were obtained from POCH Gliwice, Poland. All chemicals were of the highest analytical quality. 

### 3.2. Preparation of Aged serum Albumin in Vitro

A working solution of aged human (AHSA) and bovine (ABSA) serum albumin was prepared by dissolving protein in buffer Gly/NaOH (0.1 mol/dm^3^, pH 9.2) and maintaining at 3 °C for 4 days. Buffer Gly/NaOH at pH 9.2 was prepared from 0.1 mol/dm^3^ glycine and 0.1 mol/dm^3^ NaOH. The working solution of non-modified human (HSA) and bovine (BSA) serum albumin was prepared by dissolving protein in TRIS-HCl (0.01 mol/dm^3^, pH 7.4) buffer. TRIS-HCl buffer was prepared by the mixing of 0.1 mol/dm^3^ HCl with the appropriate amount of pure Tris(hydroxymethyl)aminomethane. Metoprolol (MET) stock solution was prepared by dissolving ligand in appropriate buffers. After each measurement, the pH of protein solution was confirmed by the use of a pH-meter (FEP20 Metler Toledo).

### 3.3. Fluorescence and UV-Vis Spectra—Second Derivative

Fluorescence and absorption spectra were recorded at 37 °C with 1 cm × 1 cm × 4 cm quartz cells. The fluorescence emission spectra were recorded on JASCO Fluorescence Spectrophotometer (Jasco International Co., Ltd., Tokyo, Japan) FP-6500 and collected in the wavelength range between 285 nm–420 nm (λ_ex_ 280 nm) and between 300 nm–420 nm (λ_ex_ 295 nm). The excitation and emission slits were set at 5 and 10 nm, respectively. Absorption spectra were recorded on JASCO V-530 spectrophotometer (Jasco International Co., Ltd.).

Fluorescence and absorption second derivative spectra were obtained in Spectra Analysis program (Spectra Manager, version 1.55.00, Jasco International Co., Ltd.) using Savitzky and Golay algorithm, second order of polynomial and 15 data points. 

The percentage of DG and DP displacement by MET from their binding sites in albumin was calculated using the following relationship [38]:

(1) $$\frac{F_{0}-F_{n}}{F_{0}}\times 100\%$$

where *F_n_* and *F*_0_ are the fluorescence of probe with serum albumin (DG-HSA, DG-AHSA, DG-BSA, DG-ABSA, DP-HSA, DP-AHSA, DP-BSA and DP-ABSA) in the presence and absence of MET, respectively. 

### 3.4. Circular Dichroism (CD) Spectra

CD spectra were recorded on Jasco model J-1500 spectropolarimeter using 1.0 mm path length cuvette at 25 °C in a thermostated Peltier cell holder with an accuracy of ±0.05 °C. All CD spectra were collected continuously at a scan speed 20 nm/min and averaged over accumulation of three spectra. The protein concentration was 1.0 × 10^−6^ mol/dm^3^ in Tris-HCl buffer (0.01 mol/dm^3^, pH 7.4). Buffer spectrum was subtracted from the sample spectrum. The observed ellipticity (*θ_obs_*) was converted to the Mean Residue Ellipticity (*θ_MRE_*) in deg × cm^2^ × dmol^−1^ using the following equation:

(2) $$\theta_{MRW}=\frac{\theta_{obs}}{10nlC_{p}}$$

where *n* is the number of amino acid residues, *l* is the path length of the cuvette, and *C_p_* is the protein concentration.

The fractional content of the secondary structure elements of the proteins was calculated in CD Multivariate SEE program using the standard calibration models. 

### 3.5. Computational Simulation

Tertiary structure of HSA and BSA required for computational simulation were downloaded from the Protein Data Bank PDB database [39] with accessions 1AO6 [40] and 4F5S [41], respectively. Theoretical structure of metoprolol (MET) was minimized and optimized by the Austin Model 1 (AM1)-semi-empirical method using the Atomic and Molecular Electronic Structure System (GAMESS) [42]. Computational simulation experiment was performed using the Piecewise Linear Potential Protein-Ligand ANT System (PLANTS_PLP_) empirical scoring algorithm [43] implemented in CLC Drug Discovery Workbench software [44]. Computational results were presented in arbitrary units and 3D graphically elaborated with the BIOVIA Discovery Studio visualizer [45].

## 4. Conclusions

Based on the analyzed data, we can conclude that the molecular rearrangements during N-A transition are affected by alkaline ageing to subdomains I, II, and III of serum albumin’s tertiary structure. The reduction of α-helical content and the increase of β structural elements of albumin indicates the formation of aggregates. It is noteworthy that at the molecular level, human serum albumin (HSA) is more sensitive to ageing than bovine (BSA) albumin, and the increase of HSA elimination is caused not only by the basic amino acid modification but also the ageing process influence on the specific albumin binding sites. These structural alterations cause MET binding affinity changes towards aged HSA. Human serum albumin N-A isomerization leads to the increase of metoprolol free fraction in serum that may cause side effects.

This study reaffirms that in elderly people and in the presence of diseases associated with ageing, the accumulation of modified proteins due to the formation of crosslinks and aggregates occurs, and the protein becomes more resistant to intracellular proteolytic systems.

## Figures and Tables

**Table 1:** Second derivative values (d^2^A/dλ^2^) for wavelength range of the protein chromophores: tryptophanyl (Trp), tyrosyl (Tyr), and phenylalanine (Phe).

λ (nm)	295	283	287	260	252
AHSA	0.0014	−0.0012	−0.0013	0.0001	0.0011
*normalized* HSA	0.0018	−0.0017	−0.0019	0.0001	0.0015
ABSA	0.0013	−0.0015	−0.0012	0.0002	0.0012
*normalized* BSA	0.0016	−0.0020	−0.0018	0.0002	0.0016

**Table 2:** Relative peak composition (parameter *H*) of albumin tryptophanyl residues at λ_ex_ 295 nm and λ_ex_ 275 nm.

	*H*_275nm_	λ_min_ (nm)	λ_max_ (nm)	*H*_295nm_	λ_min_ (nm)	λ_max_ (nm)
*normalized* HSA	0.017	311	289	0.550	385	395
AHSA	0.46	312	289	0.065	385	395
BSA	0.71	289	304	0.800	385	395
*normalized* ABSA	0.850	303	289	0.990	385	393

**Table 3:** The percentage (%) content of the secondary structure elements of HSA, AHSA, BSA, and ABSA calculated in the CD Multivariate SEE program using the standard calibration models; protein concentration was 1 × 10^−6^ mol/dm^3^.

	θ_MRE_ at 222 nm *	% α-Helix	% β-Sheet	% Turn	% Other
HSA	−25436.1	58.9	23.1	2.0	16.2
AHSA	−20892.2	49.2	13.8	4.8	32.2
BSA	−15826.2	40.9	27.9	6.5	24.8
ABSA	−17859.2	39.1	22.7	4.8	33.5

* θ_MRE_ deg·cm^2^·dmol^−1^.

**Table 4:** The percentage (%) of DG and DP displacement from their binding site in HSA, AHSA, BSA, and ABSA by MET; SA:MET molar ratio 1:10.

Molar Ratio Serum Albumin:Probe	% of Displacement			
	HSA	AHSA	BSA	ABSA
SA:DG 1:0.5	47.12	23.60	10.10	11.81
SA:DG 1:1	27.98	59.85	12.06	21.05
SA:DP 1:0.5	39.16	33.28	41.20	26.27
SA:DP 1:1	33.29	47.59	46.05	25.94
